# Single-cell RNA sequencing combined with artificial intelligence to uncover immunotherapy biomarkers and decipher drug resistance mechanisms in glioblastoma

**DOI:** 10.3389/fonc.2026.1970165

**Published:** 2026-10-05

**Authors:** Tao Fu, Mengwan Hu, Xian Wu

**Affiliations:** 1 Heilongjiang University of Chinese Medicine, Harbin, Heilongjiang, China; 2 Heilongjiang Provincial Hospital, Harbin, Heilongjiang, China; 3 The First Affiliated Hospital of Heilongjiang University of Chinese Medicine, Harbin, Heilongjiang, China

**Keywords:** artificial intelligence, biomarkers, glioblastoma, immunotherapy, single-cell RNA sequencing

## Abstract

Glioblastoma represents the most aggressive primary malignant tumor of the central nervous system, characterized by extensive intratumoral heterogeneity, profound immunosuppression, therapeutic resistance, and poor clinical outcomes. Although immunotherapy has revolutionized cancer treatment, its clinical efficacy in GBM remains limited, largely due to the complexity of the tumor ecosystem and the lack of validated predictive biomarkers. Recent advances in single-cell RNA sequencing (scRNA-seq), spatial transcriptomics, and artificial intelligence (AI)-based computational approaches have provided unprecedented opportunities to decipher tumor heterogeneity and identify clinically actionable biomarkers. This mini-review integrates studies related to scRNA-seq and its combination technology, further elaborating on the tumor plasticity, immune suppression, and metabolic-epigenetic reprogramming mechanisms of GBM. The article systematically summarizes the complete analysis process of integrating single-cell multi-omics data by AI and highlights the application value of AI in validating biomarkers. The above research ideas are expected to overcome clinical resistance problems, optimize patient stratification schemes, and enhance the efficacy of immunotherapy, providing theoretical support for developing highly stable prognostic biomarkers and achieving individualized precise immunotherapy for glioblastoma.

## 1 Introduction

Glioblastoma (GBM) is the most aggressive primary malignant tumor of the central nervous system, accounting for a substantial proportion of malignant gliomas (1). Despite continuous optimization of surgical, radiation, and temozolomide (TMZ)-based chemotherapy regimens, the treatment outcomes for GBM have not fundamentally improved, and issues of drug resistance and recurrence remain severe (2). Existing studies have gradually revealed that drug resistance and recurrence of GBM are influenced by a complex network among tumor cell plasticity, immunosuppressive microenvironments, and metabolic–epigenetic reprogramming. Besides the mechanisms mentioned above, tumor cells can also evade treatment through the complex interactions between various programmed cell death (PCD) pathways (3). Traditional bulk transcriptome sequencing has long been applied to GBM research, yet it cannot capture cell-to-cell heterogeneity, dynamic cell state transitions and complex intercellular interactions in the GBM microenvironment, which restricts the in-depth interpretation of drug resistance and tumor recurrence mechanisms (4, 5). The rapid development of single-cell RNA sequencing (scRNA-seq) and complementary technologies has broken the limitations of bulk sequencing, allowing researchers to characterize GBMs at the single-cell level (6). However, it is worth noting that scRNA-seq and complementary technologies also bring new transformation problems. Screening differential genes, cell status, or signaling pathways does not mean that it has clinical application value. Artificial intelligence (AI) provides a feasible way to solve the above problems, which can calculate the prediction results and transform them into clinical practice.

Based on this, this review places scRNA-seq within the framework of AI-driven precision oncology research. Unlike existing reviews that separately describe the application of scRNA-seq in GBM or artificial-intelligence-based research on tumor biomarkers, this review integrates these two research dimensions for further improvement. Firstly, we summarized how scRNA-seq and complementary technologies have revolutionized the understanding of tumor heterogeneity and immunosuppression in GBM. Secondly, this paper systematically elucidates three core biological mechanisms that induce GBM treatment resistance: tumor cell plasticity and drug-resistant subclonal evolution, tissue-specific immunosuppression caused by T cell depletion, and metabolic reprogramming that is bidirectionally regulated with epigenetic modifications. On this basis, this paper focuses on the application strategies of AI in screening scRNA-seq and complementary technologies characteristic markers and constructing clinical prediction models and summarizes various bottlenecks in current research so as to provide research reference for clinical translation of GBM-related prognostic biomarkers and support optimization of individualized diagnosis and treatment plans for patients.

## 2 Single-cell and spatial profiling defines the biological substrate for AI-driven biomarker discovery

ScRNA-seq is a high-throughput technology that analyzes transcriptomic characteristics at single-cell resolution. By depicting cell states and their heterogeneity, it gradually reveals complex multi-dimensional molecular features within cells (7). In cancer research, scRNA-seq has been widely used to systematically analyze the immune microenvironment of tumor cells at the single-cell level, reveal dynamic changes between cells, and identify key gene expression, thus providing an important basis for clarifying the molecular basis of tumor drug resistance and recurrence.

In addition, scRNA-seq can be combined with spatial transcriptomics (ST). By retaining tissue spatial structure information, it compensates for the inability of scRNA-seq to characterize spatial locations, providing an important tool for understanding the spatial relationship between tumor and microenvironmental cells (8). Through the integration of the two techniques, researchers can identify cell states and cell-cell interaction patterns in specific regions at spatial resolution, such as immune-excluded zones and tumor-invasive borders. This also explains why scRNA-seq combined with ST can reveal the mechanisms by which tumors adapt to new environments from a single-cell perspective (9–11).

Meanwhile, bioinformatics analysis has enhanced the ability to interpret single-cell data. Trajectory inference algorithms can reconstruct cell differentiation and developmental trajectories as well as lineage relationships (12–14), while cell-cell communication analysis can predict ligand-receptor interactions between tumor and microenvironmental cells (15). Based on the integration of multi-dimensional single-cell information, GBM is gradually being redefined as a dynamically evolving and highly interactive complex ecosystem.

However, most computational outputs generated from scRNA-seq remain exploratory. Predicted cell–cell interactions, trajectories, and pathway activities should not be interpreted as direct evidence of biological causality without experimental validation. This distinction is particularly important when single-cell findings are subsequently incorporated into AI-driven clinical prediction models. Candidate biomarkers should ideally undergo orthogonal validation using bulk transcriptomic data, spatial profiling, protein-level assays, functional experiments, or independent clinical cohorts. Without sufficient biological and clinical validation, the clinical applicability of AI models remains unclear.

## 3 Single-cell evidence reveals biological determinants of immunotherapy and treatment resistance

### 3.1 Tumor cell plasticity and subclonal evolution

One of the core characteristics of GBM drug resistance is the high transcriptional plasticity of malignant cells. Single-cell transcriptomic analysis has identified multiple tumor cell transcriptional states, mainly including neural progenitor-like (NPC-like), oligodendrocyte progenitor-like (OPC-like), astrocyte-like (AC-like), and mesenchymal-like (MES-like) cell populations (16, 17). Notably, these states are not static but show significant plasticity and can dynamically switch between different transcriptional states. Experimental studies have suggested that treatment-induced transcriptional reprogramming may promote the transition of GBM cells toward highly proliferative AC/MES-like states, which are associated with increased invasiveness and treatment resistance (17).

Subclonal evolution provides another layer of therapeutic adaptation. Christine et al. used scRNA-seq and experimental models to investigate GBM cells exposed to RTK inhibition and identified resistant subclones characterized by IRS1/IRS2 amplification and persistent AKT activation (18). Similarly, Sied Kebir et al. identified TMZ-induced ALDH1A1^+^ subclones associated with acquired resistance and recurrence through AKT-related signaling (19). Together, these findings suggest that GBM drug resistance may arise through both transcriptional plasticity and the selective expansion of subclones with genetic or epigenetic advantages. More recent single-cell and multi-omics studies have further suggested that therapeutic pressure may promote neuroepithelial-to-mesenchymal-like transitions through AP1-associated chromatin remodeling, accompanied by selective expansion of resistant subclones (20). Tumor resistance should be viewed as a dynamic process involving both cellular state transitions and clonal selection. Furthermore, transcriptional plasticity and the subclonal evolution at the genetic level are not mutually exclusive but may jointly shape the biological processes underlying treatment resistance and recurrence in GBM (17). The above mechanisms are illustrated in **Figure 1**.

**Figure 1:**
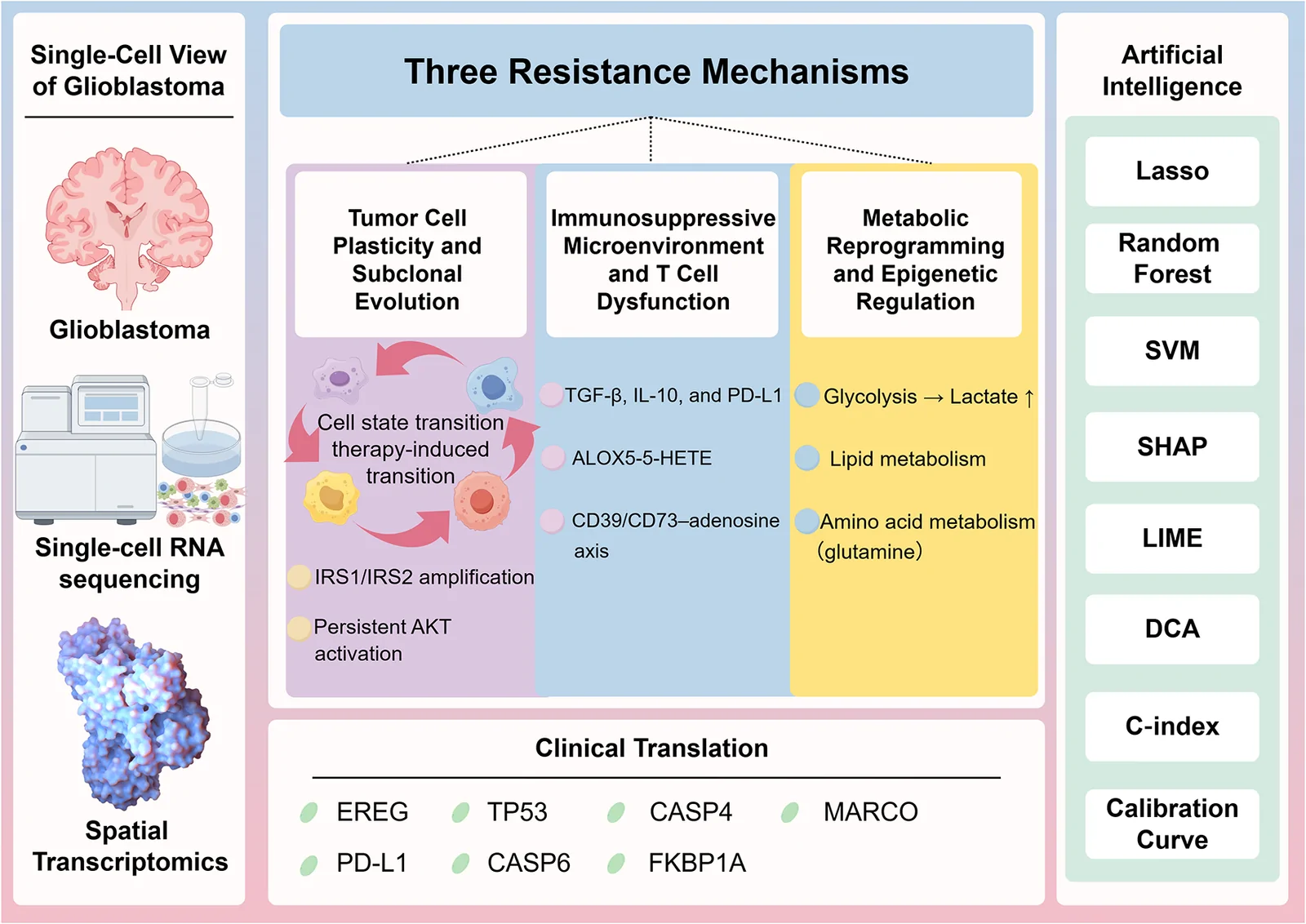
Schematic diagram showing the immune resistance mechanism of GBM from a single-cell perspective and the artificial intelligence-driven precise treatment approach.

From a biomarker perspective, this heterogeneity has important implications. A single gene may inadequately represent a dynamic tumor state, whereas a composite AI-derived signature incorporating transcriptional state, clonal characteristics, and clinical variables may provide a more robust representation of treatment-resistant disease. Such features may help to identify patients with high risk of recurrence or poor clinical prognosis and also provide hypotheses for potential therapeutic targets. Representative scRNA-seq studies of GBM are summarized in **Table 1**.

**Table 1:** Representative scRNA-seq studies on GBM drug resistance.

Study	Publication year	Disease or sample context	Single-cell method	Principal finding	Therapeutic implication(experimentally supported or hypothetical)
Neftel et al. 31327527	Aug 8, 2019	This study combined scRNA-seq of 28 GBM, 401 TCGA GBM samples and functional/lineage tracing to investigate GBM cell states, genetics and plasticity.	Used SMART-Seq2, 10× scRNA-seq, CNA inference, barcoding lineage tracing and RNA-ISH to dissect GBM cell states and plasticity.	Identified four major GBM cellular states (NPC-like, OPC-like, AC-like, MES-like) and demonstrated dynamic state transitions. Revealed tumor cell plasticity as a driver of therapy resistance	experimentally supported
Eyler et al. 32669109	Jul 15, 2020	Tumors adapt to treatment via transient or stable genetic/epigenetic changes driving intratumoral heterogeneity. Single-cell tools are needed to trace adaptive resistant cells. RTK inhibitors fail in GBM due to either genetic heterogeneity or epigenetic bypass of targeted signaling.	Lentiviral lineage barcoding, scRNA-seq, low-coverage WGS for clonal copy number validation.	Lineage tracing combined with scRNA-seq revealed emergence of IRS1/IRS2-amplified resistant subclones with AKT activation. Demonstrated that subclonal evolution contributes to acquired resistance	experimentally supported
Chen et al. 39142719	Aug 13, 2024	Oxylipin metabolism plays an essential role in glioma progression and immune modulation in the tumor microenvironment. Lipid metabolic reprogramming has been linked to macrophage remodeling, while the understanding of oxylipins and their catalyzed enzymes lipoxygenases in the regulation of glioma-associated microglia/macrophages remains largely unexplored.	Flow cytometry, single cell level immunofluorescence staining	ALOX5-driven M2-like polarization of TAMs increased PD-L1 expression Linked TAM-mediated immunosuppression to tumor progression	experimentally supported
Coy et al. 35973991	August 16, 2022	The mechanism by which the immune microenvironment of glioma promotes tumor occurrence remains unclear. In this study, using scRNA-seq and multiplexed tissue imaging techniques, the composition, spatial distribution, and clinical significance of extracellular purine signaling pathways in glioma were characterized.	scRNA-seq, multi-round cycle immunofluorescence CyCIF tissue single-cell spatial imaging, flow sorting to obtain immune single cells, spatially resolved mass spectrometry imaging for single-cell metabolic detection.	Integrated single-cell and spatial analyses identified CD39/CD73-mediated purinergic signaling in immune-excluded niches Revealed spatially organized immune suppression within GBM	experimentally supported
Han et al. 41275140	Nov 22, 2025	GBM is the most aggressive adult brain tumor, marked by intratumoral heterogeneity and therapy resistance. Metabolic reprogramming through histone lactylation has been linked to tumor progression and immune suppression. However, the spatial and single-cell landscape of lactylation in GBM and its prognostic significance remain poorly understood.	scRNA-seq, InferCNV for malignant cell identification, SCENIC for transcriptional regulation analysis, CellChat, Monocle + CytoTRACE for pseudo-temporal trajectory, scPagwas for single-cell phenotypic association analysis.	Glycolysis-derived lactate promoted histone lactylation and immunosuppressive transcriptional programs Linked metabolic reprogramming to epigenetic regulation and resistance	experimentally supported
Li et al. 41023045	September 29, 2025	Abstract Fatty acid metabolism is critically involved in GBMpathogenesis; however, its regulatory mechanisms remain incompletely understood. In this study, we identified biliverdin reductase A (BLVRA) as a novel metabolic driver and prognostic biomarker in GBM by integrating bulk and scRNA-seq with *in vitro* functional validation.	10x scRNA-seq, Seurat quality control and Harmony batch correction, Louvain clustering, SingleR cell annotation, DoubletFinder for removing tumor cell doublets, inferCNV copy number variation analysis, CytoTRACE differentiation potential assessment, CellChat cell communication, GSVA pathway activity scoring.	The FAMRGPI model for fatty acid metabolism was constructed, confirming that BLVRA is an independent poor prognostic marker for GBM. It maintains redox homeostasis and promotes tumor proliferation and an immunosuppressive microenvironment through regulating fatty acid metabolism and is a potential metabolic therapeutic target for GBM.	3

### 3.2 Immunosuppressive microenvironment and T cell dysfunction

GBM is often described as an immune-cold tumor, but it does not completely lack T cell infiltration. T cells infiltrated into the lesion are mostly in a state of functional exhaustion, and it is difficult to exert effective anti-tumor effects in a highly immunosuppressive microenvironment (21). Tumor-associated macrophages (TAMs), including resident microglia and macrophage populations, represent major components of the GBM immune microenvironment (22, 23). TAMs may polarize toward an immunosuppressive phenotype and secrete related cytokines to inhibit anti-tumor immune responses. Chen et al. linked ALOX5–5-HETE pathway activity to M2-like polarization of GBM-associated astrocytes/macrophages and increased PD-L1 expression, suggesting that this pathway may contribute to tumor immune evasion (24). In addition, TGF-β, IL-10, and PD-L1 signaling have been linked to impaired CD8^+^ T-cell activity (25).

T-cell dysfunction is an important feature of immune suppression in GBM. Dysfunctional T cells often show reduced proliferation and cytotoxic activity, accompanied by impaired cytokine production (26, 27). Woroniecka et al. reported that CD8^+^ T cells in GBM displayed prominent exhaustion features, including increased expression of PD-1, TIM-3, and LAG-3 and decreased production of IL-2, TNF-α, and IFN-γ (28).

Spatial analyses have shown that immune-cell distribution varies considerably across different regions of GBM. In particular, immune exclusion in the tumor core and the presence of immunosuppressive niches may restrict effective immune surveillance (29). The CD39/CD73–adenosine axis has also been associated with immunomodulatory regions in GBM (30).

These findings support the development of AI-derived immune-related biomarker signatures. Future models could move beyond PD-L1 expression or individual immune-cell markers by incorporating the abundance and functional states of TAMs and exhausted T cells, together with immune-exclusion patterns, checkpoint molecules, and spatial organization of immune cells. Such composite signatures may more accurately capture the overall immunological state of individual tumors and potentially improve patient stratification for immunotherapy. It is worth noting that these markers are currently mainly used as prognostic biomarkers; only through continuous formal validation can it be confirmed whether they truly have the ability to predict the response to immunotherapy.

### 3.3 Metabolic reprogramming and epigenetic regulation

Accumulating evidence suggests that metabolic reprogramming and epigenetic remodeling are associated with GBM therapeutic resistance and may contribute to treatment adaptation. Tumor cells can achieve metabolic adaptation by reprogramming glucose, lipid, and amino acid metabolic patterns; resist treatment pressure; and maintain malignant proliferation and thus continue to survive and progress in the harsh TME (31–35). In recent years, scRNA-seq and ST studies have further shown that metabolic heterogeneity within tumors was closely related to the treatment resistance and immune microenvironment remodeling of GBM.

Han Rui et al. reported that enhanced glycolysis and lactate accumulation were associated with histone lactylation-related transcriptional changes and may contribute to immunosuppression, tumor progression, and drug resistance (36). Li Lang et al. developed a fatty-acid-metabolism-related model and identified BLVRA as an independent prognostic marker associated with redox regulation, tumor proliferation, and an immunosuppressive microenvironment (37). In contrast, He Songjiang’s team reported that enhanced glutamine metabolism was associated with increased TCA cycle activity, nucleotide synthesis, and cell-cycle progression, as well as T-cell exhaustion and immunosuppression. Interestingly, tumors characterized by a high glutamine-metabolism phenotype were predicted to be more sensitive to several therapeutic modalities (38).

Furthermore, there is a bidirectional regulatory relationship between metabolic reprogramming and epigenetics. The evidence supporting this interaction mostly comes from comprehensive cancer multi-omics studies, while the evidence for GBM is insufficient (39). Epigenetic regulation is an important driving mechanism of GBM therapeutic resistance (40), mainly including DNA methylation, histone modification, chromatin remodeling, and non-coding RNA molecules. Tumor metabolic reprogramming can inhibit immune cell function, while epigenetic reprogramming further stabilizes the immunosuppressive state.

However, many currently proposed metabolic–epigenetic relationships remain based primarily on computational association. Therefore, candidate biomarkers derived from these pathways require independent molecular and functional validation before clinical application.

## 4 Clinical translation: from computational biomarkers to treatment decisions

Based on the tumor plasticity, immunosuppressive microenvironment, metabolic epigenetic reprogramming, and drug resistance mechanisms revealed by the above single-cell studies, the ultimate value of further mining biomarkers through AI is to transform basic biological findings into operable clinical decisions. At present, single-cell studies have identified numerous candidate biomarkers and, in some cases, potential therapeutic targets supported by functional evidence. Among them, the abnormal high expression of immunomodulatory molecules such as EREG (41) and PD-L1 (42) is closely related to the immunosuppressive cell status of GBM. TP53, CASP6, CASP4 and other necroinflammation-related genes have demonstrated prognostic value, while their utility as predictive biomarkers for immunotherapy response remains unconfirmed, even though some may represent potential therapeutic targets (43). Functional studies have provided experimental evidence that molecules such as FKBP1A and MARCO may represent potential therapeutic targets and therapy-resistance phenotypes in GBM (44).

### 4.1 Standardized evaluation criteria for clinical prediction models

For the constructed prognostic and immunotherapy efficacy prediction models, it is necessary to systematically assess the potential bias risks during the model development and validation process and evaluate the model’s stability through appropriate internal validation. Further external validation should be conducted in independent cohorts to test its predictive performance, generalization ability, and clinical applicability (45, 46). The model performance can use the concordance index (C-Index) to evaluate the ability to distinguish survival outcomes, use time-dependent AUC to evaluate the predictive performance at a specific time point, and evaluate the consistency between the predicted risk and the actual clinical outcome through the calibration curve (47, 48). Decision curve analysis (DCA) can further evaluate the potential net benefit of the model in clinical decision-making (49). It is worth noting that prognostic markers are not equivalent to targeted intervention targets (50). Some of the molecular characteristics screened by AI have not been verified by perfect cell and animal function experiments and large sample clinical verification, which can only be used as an auxiliary reference for clinical evaluation.

### 4.2 Integration of AI and single-cell multi-omics for the construction of clinical biomarkers

In scRNA-seq analysis, computational frameworks such as Seurat provide standardized workflows for quality control, normalization, dimensionality reduction, clustering, and cell-state annotation, while algorithms such as Harmony can mitigate batch-associated technical variation (51, 52). At the same time, trajectory inference and intercellular communication analysis methods such as Monocle, Slingshot and CellChat can further analyze cell state transition and potential intercellular signal networks (53). It should be noted that the cell state, molecular characteristics and intercellular communication information extracted from scRNA-seq can be used as an important input for subsequent machine learning models for biomarker discovery and clinical prediction.

On this basis, machine-learning methods can be applied to identify key variables associated with clinical outcomes from high-dimensional single-cell features and further construct predictive models. LASSO regression, random forest (RF), and support vector machine (SVM) represent different strategies for feature selection and predictive modeling. LASSO realizes variable selection through L1 regularization and is suitable for high-dimensional, small-sample molecular data (54). RF is capable of capturing complex nonlinear relationships and variable interactions (55). SVM is well-suited for high-dimensional data and can build nonlinear decision boundaries via kernel functions (56). These methods have different characteristics in feature screening ability, model complexity and interpretability. Of course, there are also other more useful models available (57–59). It is worth emphasizing that the main functions of LASSO, RF and SVM are feature screening and predictive modeling, and should not be directly equated with explainable artificial intelligence (XAI).

The XAI method can be further used to explain the prediction basis of complex models. Shapley Additive Explanations (SHAP) and Local Interpretable Model-agnostic Explanations (LIME), can estimate the contribution of a single input feature to the model prediction results, thereby identifying the molecular, cellular, imaging or clinical features that drive risk prediction (60, 61). These methods are helpful to identify key genes, cell status, metabolic processes or clinical variables that drive model predictions, but their interpretation results still depend on the original model and data structure. Especially when the features are highly correlated, the independent contribution of individual variables should be interpreted with caution. Therefore, the role of XAI is mainly to improve the transparency and interpretability of the model, and cannot replace the biological mechanism verification.

Finally, it is worth noting that most currently AI-derived biomarkers in GBM are prognostic biomarkers. If they lack sufficient clinical and functional validation, can only serve as clinical auxiliary reference indicators and cannot directly guide individualized treatment.

## 5 Limitations, challenges and future perspectives

Single-cell sequencing and AI technologies have provided new research approaches for precise immunotherapy of GBM, but there are still many shortcomings that limit their clinical application.

In single-cell technology research, GBM has extremely strong spatial heterogeneity, and the clinical sampling range is limited, which easily leads to selection bias and makes it difficult to fully reflect the true biological characteristics of the tumor. The sequencing platforms and experimental procedures of different studies are not unified, and the batch effect is obvious, making it difficult to verify and reuse the analysis results of cell typing and cell interactions. At the same time, single-cell sequencing requires dissociation of tissues, losing the original spatial distribution information of cells, and cannot reveal the intrinsic relationship between tumor spatial structure and treatment resistance. In addition, the sequencing technology has high noise and limited gene capture ability, which easily misses low-abundance genes and rare drug-resistant cell subpopulations, resulting in false positive results. From a clinical perspective, the high cost of single-cell multi-omics sequencing makes it a major obstacle for its clinical routine application. Currently, scRNA-seq is mostly limited to research settings and cannot be widely promoted as a routine clinical testing method. These issues have restricted the transformation of such highly promising scientific achievements into clinical practice. The high cost of testing and the scarcity of clinical samples also further restrict the implementation of the technology.

In terms of the construction of AI models, there are generally problems of small sample size and high feature dimensions in GBM research, making the models prone to overfitting and poor external generalization performance. Most current prediction models are established based on retrospective data and lack standardized multicenter and prospective cohort validations, resulting in insufficient clinical reliability. At the same time, traditional intelligent models have poor interpretability and are difficult to elucidate the regulatory role of key cells and molecular characteristics on the prediction results. More critically, most AI markers only have statistical correlations and lack biological function validation, making it impossible to distinguish between ordinary prognostic indicators and truly predictive markers that can guide immunotherapy, greatly limiting the clinical application value of the models.

Future research can integrate multi-dimensional information such as single-cell transcriptomics, spatial transcriptomics, imaging, pathology, and clinical data. By combining interpretable AI algorithms and standardized experimental systems, it can optimize detection costs, screen core representative molecules, and conduct strict validations based on prospective clinical cohorts. In addition to omics analysis techniques, emerging CRISPR-Cas-based molecular biosensing platforms offer broad research prospects for detecting tumor-associated nucleic acid signatures, including gene mutations, DNA methylation, fusion transcripts, and microRNAs (62–65). These platforms can be further synergized with microfluidics, nanomaterials, and artificial-intelligence-driven signal-decoding technologies, enabling rapid and low-cost biomarker detection workflows. This will break through the limitations of existing technologies and models and promote the transformation of new biomarkers for GBM from basic research to individualized precise immunotherapy.

## 6 Conclusion

GBM remains one of the most lethal intracranial malignant tumors worldwide, characterized by extensive intratumoral heterogeneity, widespread treatment resistance, and high recurrence rate. Treatment failure is associated with tumor-cell transcriptional plasticity, an immunosuppressive microenvironment, and metabolic–epigenetic reprogramming. ScRNA-seq has provided important insights into resistance mechanisms in GBM. AI and machine learning algorithms serve as the key bridge connecting basic single-cell omics research and clinical individualized treatment. Future studies combining multi-omics and AI modeling with prospective clinical validation may enable the development of robust prognostic biomarkers and, where supported by treatment-specific clinical evidence, predictive biomarkers of immunotherapy response, thereby promoting more precise and individualized immunotherapy for GBM.
